# 1395. The Path of Healing: Demographic Features and Therapeutic Trajectory of Patients with American Tegumentary Leishmaniasis in a City of Northern Brazil

**DOI:** 10.1093/ofid/ofad500.1232

**Published:** 2023-11-27

**Authors:** Alana Pinheiro Alves, Francisco Alrimar Silva Xavier, Gustavo Alessandro de Sousa Pereira, Fernanda Jacqueline Teixeira Cardoso, Andrea Leite de Alencar Salgado, Nadia Vicencia do Nascimento Martins

**Affiliations:** Western Michigan University, Kalamazoo, Michigan; Universidade do Estado do Para, Santarem, Para, Brazil; Universidade do Estado do Para, Santarem, Para, Brazil; Universidade do Estado do Para, Santarem, Para, Brazil; Universidade do Estado do Para, Santarem, Para, Brazil; Universidade do Estado do Para, Santarem, Para, Brazil

## Abstract

**Background:**

American Tegumentary Leishmaniasis (ATL) is a neglected infectious disease caused by protozoa of the *Leishmania* genus. Its incidence in the state of Para, Northern Brazil, reached 38,95 cases per 100,000 individuals in 2017. During this illness, people will move through several layers of a healthcare structure on a path for solutions to their affliction. This is described as therapeutic trajectory or itinerary, and it is directly influenced by determinants of health. In this scenario, we question what the trajectory of patients with ATL within healthcare in the city of Ruropolis, Brazil, is.

**Methods:**

This was a transversal retrospective study. Qualitative data was also obtained through patient interviews. The research was developed in the city of Ruropolis, in Northern Brazil, and included individuals who received care through the Brazilian Unified Health System (SUS). All patients or responsible adults involved in the research signed an informed consent form.

**Figure:**
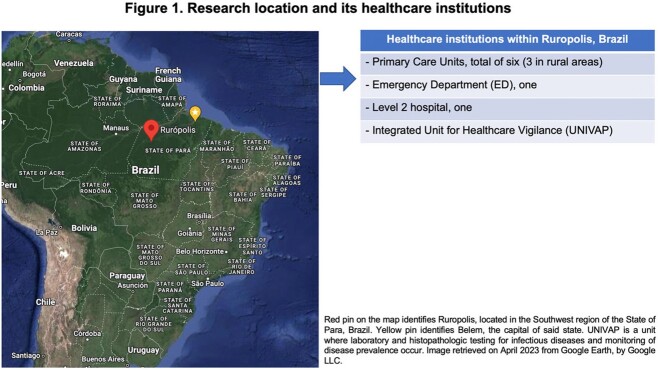

**Figure:**
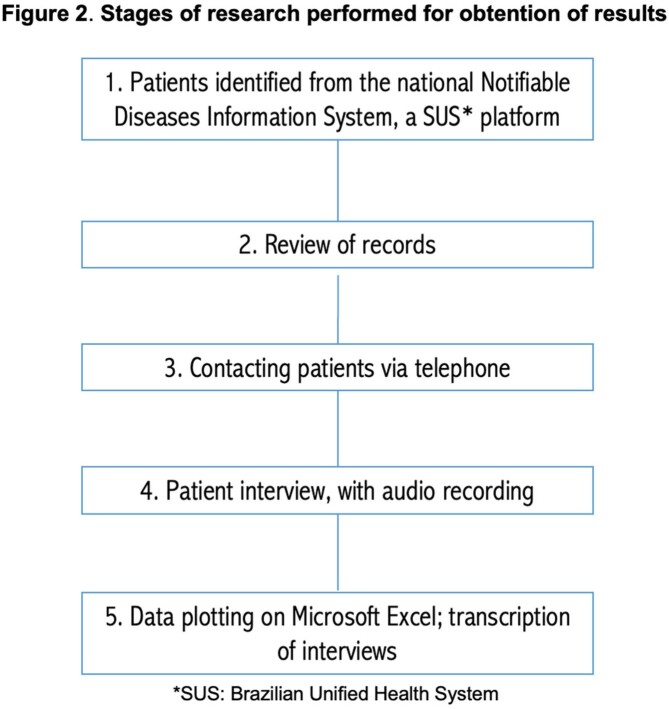

**Results:**

A total of 49 patients were initially included, but only 27 were interviewed. In 2021, ATL was diagnosed and notified every month, although April had the highest incidence (12 new cases). Over half of the patients resided in rural areas, and most worked in agriculture. Clear differences in trajectory were noted between patients or urban versus rural areas, as the latter mostly visited the local Emergency Department (ED) for care. Histopathologic work-up of all patients as performed. Regarding treatment, individuals had to relocate up to 50 kilometers daily to receive injections. Home healthcare services were not available.

Overall, 23 patients received intravenous treatment with meglumine antimoniate, and 10 had a myriad of effects, such as fatigue, peripheral edema, and vertigo. Only four patients had intralesional therapy. Of 27 patients, 22 did not know how to prevent ATL or refused to answer.

**Figure:**
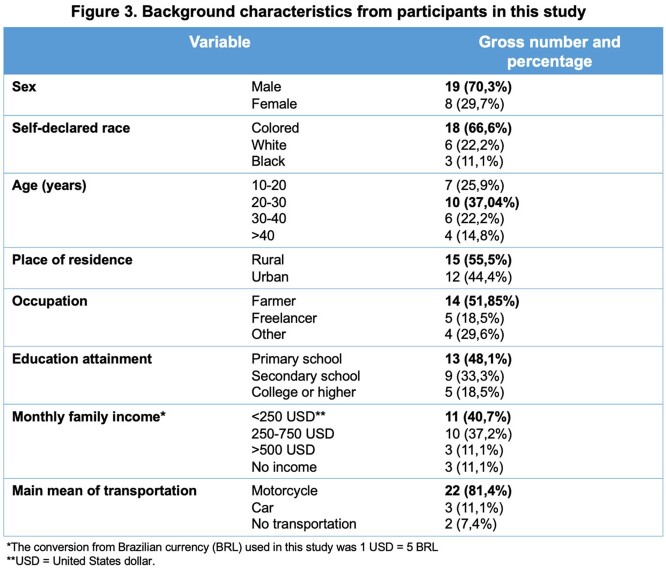

**Figure:**
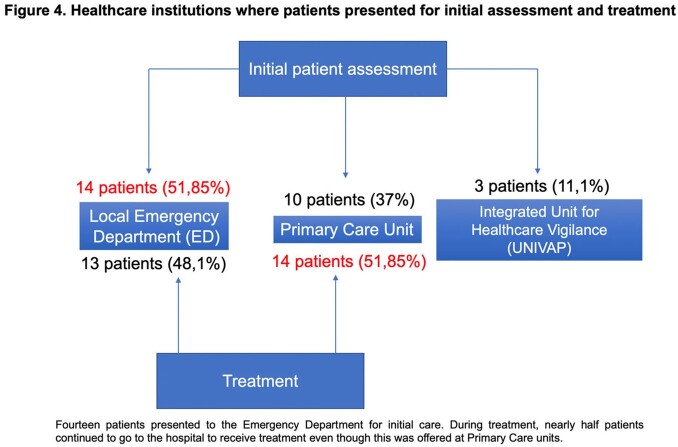

**Figure:**
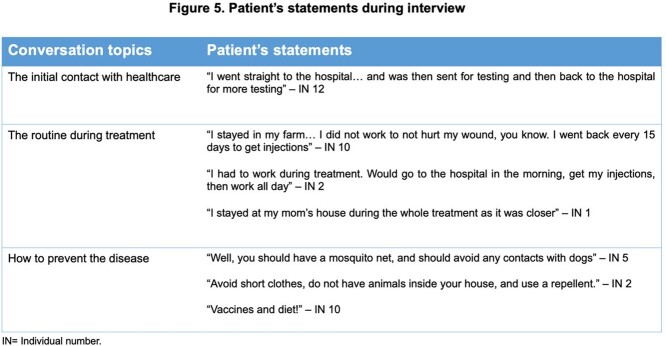

**Conclusion:**

The struggles faced by patients with ATL are diverse. The local population is unaware of the different layers of service within healthcare, and a hospital-centric model seems to prevail. Patients seek care wherever it seems appropriate to solve their trouble. The narratives on knowledge of the disease reveal the need for further health education, preferably with the introduction of active teaching methods.

**Disclosures:**

**All Authors**: No reported disclosures

